# Serum Chemerin Concentrations Associate with Beta-Cell Function, but Not with Insulin Resistance in Individuals with Non-Alcoholic Fatty Liver Disease (NAFLD)

**DOI:** 10.1371/journal.pone.0124935

**Published:** 2015-05-01

**Authors:** Erifili Hatziagelaki, Christian Herder, Anastasia Tsiavou, Tom Teichert, Athina Chounta, Peter Nowotny, Giovanni Pacini, George Dimitriadis, Michael Roden

**Affiliations:** 1 2^nd^ Department of Internal Medicine, Research Institute and Diabetes Center, Athens University, “Attikon” University General Hospital, Athens, Greece; 2 Institute of Clinical Diabetology, German Diabetes Center, Leibniz Center for Diabetes Research at Heinrich Heine University Düsseldorf, Düsseldorf, Germany; 3 German Center for Diabetes Research, Partner Düsseldorf, Düsseldorf, Germany; 4 4^th^ Department of Internal Medicine, “Attikon” University General Hospital, Athens, Greece; 5 Metabolic Unit, CNR Neuroscience Institute, National Research Council, Padova, Italy; 6 Department of Endocrinology and Diabetology, University Hospital Düsseldorf, Düsseldorf, Germany; Scientific Directorate, Bambino Hospital, ITALY

## Abstract

The novel adipokine chemerin has been related to insulin-resistant states such as obesity and non alcoholic fatty liver disease (NAFLD). However, its association with insulin resistance and beta cell function remains controversial. The main objective was to examine whether serum chemerin levels associate with insulin sensitivity and beta cell function independently of body mass index (BMI), by studying consecutive outpatients of the hepatology clinics of a European university hospital. Individuals (n=196) with NAFLD were stratified into persons with normal glucose tolerance (NGT; n=110), impaired glucose tolerance (IGT; n=51) and type 2 diabetes (T2D; n=35) and the association between serum chemerin and measures of insulin sensitivity and beta cell function as assessed during fasting and during oral glucose tolerance test (OGTT) was measured. Our results showed that serum chemerin positively associated with BMI (P=0.0007) and C peptide during OGTT (P<0.004), but not with circulating glucose, insulin, lipids or liver enzymes (all P>0.18). No BMI independent relationships of chemerin with fasting and OGTT derived measures of insulin sensitivity were found (P>0.5). Chemerin associated positively with fasting beta cell function as well as the OGTT derived insulinogenic index IGI_cp and the adaptation index after adjustment for age, sex and BMI (P=0.002-0.007), and inversely with the insulin/C peptide ratio (P=0.007). Serum chemerin neither related to the insulinogenic index IGI_ins nor the disposition index. In conclusion, circulating chemerin is likely linked to enhanced beta cell function but not to insulin sensitivity in patients with NAFLD.

## Introduction

Chemerin is highly expressed in white adipose tissue, liver and lungs, secreted as inactive prochemerin and binds after activation to the chemokine—like receptor 1 (CMKLR1) [1,2]. Binding of chemerin to CMKLR1 in immune cells and adipose tissue stimulates chemotaxis at sites of inflammation [3,4].

Previous studies in humans suggested that chemerin is increased and/or associates with age [3,5], degree of adiposity based on body mass index (BMI) or fat mass and obesity-related markers such as leptin and resistin [3,6–10]. Circulating chemerin has been also related to degree of glycemia and insulin resistance based on fasting glucose, glycated hemoglobin (HbA1C) and fasting insulin [3,5,6,7,8]. Furthermore, chemerin has been positively associated with blood pressure [3,7,8] and biomarkers of inflammation, such as C-reactive protein (CRP) [3,5,8,9]. In line, body weight reduction by lifestyle intervention [11] and bariatric surgery [12,13] also reduced serum chemerin levels. Employing different measures of insulin sensitivity yielded contradictory results regarding its association with chemerin levels [14–27]. In addition, data on a relationship between chemerin and beta-cell function are scarce in humans. In chemerin-deficient mice, chemerin positively regulates glucose-dependent insulin secretion [28]. Likewise, circulating chemerin tended to be positively associated with HOMA-β as a surrogate marker of fasting insulin secretion in Japanese patients with metabolic syndrome or type 2 diabetes [20].

Circulating levels of chemerin have been also associated with non-alcoholic fatty liver disease (NAFLD) [13,29,30], which comprises a broad spectrum of disorders ranging from simple fatty liver to nonalcoholic steatohepatitis (NASH) and cirrhosis [31]. NAFLD is closely linked to obesity and insulin resistance, which frequently co-exists with impaired glucose tolerance (IGT) or type 2 diabetes [32,33].

As chemerin may modulate insulin resistance and inflammatory responses [34], we examined its role in patients with NAFLD. More specifically, we tested (i) whether chemerin levels differently associate with insulin sensitivity and/or beta-cell function using both fasting levels and dynamic changes of glucose and insulin after oral glucose loading and (ii) if so, whether these associations occur independently of BMI in patients with NAFLD and different degrees of glucose tolerance.

## Materials and Methods

### Study participants

We studied consecutive patients who presented with elevated serum aminotransferases at the outpatient Hepatology Clinics of Attikon University General Hospital in Athens, Greece, between June 2009 and May 2012, and who were diagnosed with NAFLD. The diagnosis of NAFLD was based on the presence of increased liver transaminases, i.e. alanine transaminase (ALT) and gamma-glutamyl transpeptidase (γ-GT) 1.5-2times above the upper limit of the normal range, along with typical hepatic fat infiltration, i.e. “bright liver” or hyperechogenic appearance employing abdominal ultrasound and the exclusion of other possible causes of liver disease, including alcoholic liver disease (alcohol consumption exceeding 20 g/day), adverse drug reactions, viral hepatitis, autoimmune disorders and hereditary diseases affecting the liver [35].

Upon inclusion in the study, height and weight from each patient were measured. Systolic and diastolic blood pressure values were recorded. After overnight fasting, the patients with NAFLD underwent fasting blood sampling and a 75-g oral glucose tolerance test (OGTT), during which blood samples were obtained through an indwelling peripheral vein cannula at 0, 30, 60, 90 and 120 min to measure plasma glucose, serum insulin and serum C-peptide concentrations. Patients with known history of diabetes or a fasting plasma glucose ≥126 mg/dl did not undergo an OGTT and were therefore excluded from the study. A total of 197 patients were enrolled in the study and classified according to the criteria by the American Diabetes Association [36] into 110 individuals with normal glucose tolerance (NGT), 52 with impaired glucose tolerance (IGT) and 35 with type 2 diabetes (T2D).

### Ethics statement

The institutional review board of “Attikon” University General Hospital approved this study and the patients gave their informed consent in writing.

### Laboratory analyses

Serum cholesterol (total, LDL, HDL), triglycerides, aspartate transaminase (AST), ALT and γ-GT were assessed using a Cobas 8000 analyzer (Roche, Basel, Switzerland). Plasma glucose was measured using the glucose oxidase method (YSI, Yellow Springs Instruments, Yellow Springs, CO). Serum C-peptide and insulin levels were quantified using the respective radioimmunoassays from Millipore (St. Charles, MO). Serum chemerin was measured by an enzyme-linked immunosorbent assay (Chemerin Human ELISA; Biovendor, Brno, Czech Republic) with intra- and inter-assay coefficients of variation of 3.4% and 6.4%, respectively.

### Calculation of insulin sensitivity and secretion

In the fasted state, insulin sensitivity was assessed from QUICKI = 1/[log (insulin) + log (glucose)] [38], while beta-cell function was estimated with the ratio of insulin to glucose concentration. During the OGTT, total and incremental areas under the time curves (AUC) of plasma glucose, insulin and C-peptide concentrations were calculated using the trapezoidal rule. The oral glucose insulin sensitivity (OGIS) represents a measure of dynamic insulin sensitivity as previously described and validated against the hyperinsulinemic-normoglycemic clamp [37,38]. Beta-cell function was assessed from insulinogenic indices (IGI) [39]. The IGI_cp, given as the ratio of the difference between C-peptide at 30 min and at fasting versus the analogous difference for glucose at 30 min and at fasting, provides an empirical index mirroring beta-cell function at portal level. Using insulin instead of C-peptide yields the classic IGI_ins [39]. The beta-cell adaptation index reflects the mechanism, by which the beta-cell modulates insulin release in response to changes of insulin resistance, and is given as OGIS times IGI_cp, while the disposition index is given as OGIS times IGI_ins [38].

### Statistical analysis

The characteristics of the study sample are given as percentages for categorical variables, mean and standard deviation (SD) for continuous variables with normal distribution and median (25^th^ and 75^th^ percentiles) for continuous variables without normal distribution. The Kolmogorov-Smirnov test was used to test for normal distribution of data. Group comparisons for these variables were performed using Fisher’s exact test, t-test or ANOVA with Dunnett’s multiple comparison test (for two or more groups) and Kruskal-Wallis test with Dunn’s multiple comparison test for more than two groups, respectively. Correlations were assessed as partial Spearman correlation coefficients adjusting for the variables indicated. P values lower than 0.05 were considered statistically significant. Statistical analyses were performed using GraphPad Prism version 6.02 (GraphPad Software, La Jolla, CA) and SAS version 9.3 (SAS Institute, Cary, NC).

## Results

### Anthropometric and metabolic characteristics

Table 1 presents the basic characteristics of the study population stratified by glucose tolerance status. Except from younger age in NGT compared with IGT and T2D, the groups did not differ with respect to sex, BMI as well as serum lipids and transaminases.

**Table 1:** Basic Characteristics of Study Participants.

Variable	Normal glucose tolerance	Impaired glucose tolerance	Type 2 diabetes
n (% men)	110 (51)	51 (47)	35 (54)
Age, years	47 ± 11	53 ± 10**	57 ± 11**
BMI, kg/m²	29 ± 6	30 ± 4	31 ± 5
**Serum lipids**			
Triglycerides, mg/dl	109 (79; 146)	121 (83; 175)	112 (92; 191)
Total cholesterol, mg/dl	207 ± 42	209 ± 41	205 ± 51
HDL cholesterol, mg/dl	54 ± 18	51 ± 25	45 ± 12
LDL cholesterol, mg/dl	133 ± 37	123 ± 31	129 ± 39
**Liver enzymes**			
AST, U/l	29 (21; 44)	31 (22; 47)	32 (24;45)
ALT, U/l	48 (30; 75)	53 (31; 83)	53 (31; 74)
γ-GT, U/l	57 (26; 127)	56 (32; 121)	57 (26;131)
**Measures of glycaemia**			
Fasting glucose, mg/dl	82 ± 11	88 ± 13*	107 ± 25**
2-h glucose, mg/dl	107 ± 20	167 ± 18**	249 ± 39**
AUC glucose, g/l*2h	159 ± 30	206 ± 25**	259 ± 41**
Incremental AUC glucose, g/l*2h	61 ± 25	100 ± 21**	131 ± 20**
**Insulin and C-peptide levels**			
Fasting insulin, μU/ml	12.3 (9.1; 16.6)	12.3 (9.3; 16.4)	17.8 (12.7; 25.1)**
AUC insulin, U/l*2h	9.9 (6.4; 13.3)	11.7 (8.0; 18.8)	9.7 (7.3; 15.1)
Incremental AUC insulin, U/l*2h	8.4 (5.4; 11.9)	10.2 (6.8; 16.0)	7.8 (5.6; 12.1)
Fasting C-peptide, ng/ml	0.30 (0.10; 0.94)	0.32 (0.10; 1.00)	0.49 (0.10; 1.00)
AUC C-peptide, mg/l*2h	0.11 (0.03; 0.51)	0.11 (0.02; 0.44)	0.15 (0.02; 0.36)
Incremental AUC C-peptide, mg/l*2h	0.07 (0.01; 0.44)	0.07 (0.01; 0.32)	0.08 (0.01; 0.27)
**Measures of insulin sensitivity**			
QUICKI	0.40 ± 0.04	0.39 ± 0.03	0.36 ± 0.04**
OGIS, ml/min*m²	420 ± 82	358 ± 72**	316 ± 71**
**Measures of beta-cell function**			
Fasting beta-cell function	22 (8; 74)	24 (8; 64)	35 (10; 58)
Insulinogenic index IGI_ins, nmol/mmol	15.1 (11.1; 23.2)	10.4 (7.0; 15.7)**	7.1 (4.1; 12.6)***
Insulinogenic index IGI_cp, nmol/mmol	9.6 (1.2; 41.8)	4.1 (0.6; 18.0)*	3.7 (0.4; 13.6)*
Disposition index	2.7 (2.1; 3.7)	2.2 (1.6; 3.2)**	1.4 (0.9; 2.3)***
Adaptation index	2.1 (0.5; 8.3)	1.5 (0.3; 4.1)	1.0 (0.2; 4.1)*
Insulin/C-peptide, molar ratio	0.54 (0.21; 2.12)	0.58 (0.28; 1.91)	0.48 (0.26; 2.05)

Data are missing for 16–22% of the total study population for the lipid parameters, for 10–11% for the liver enzymes and for 6% for C-peptides and derived indices.

*P<0.05,

**P<0.01,

***P<0.001 compared to controls.

Individuals with IGT and T2D had higher fasting, 2-h and AUC glucose than individuals with NGT (Table 1). Fasting insulin was higher in T2D than in NGT, while other insulin and C-peptide-related parameters were not different between groups. QUICKI was lower in T2D than in IGT and NGT, while OGIS was lower in both T2D and IGT than in NGT (Table 1). Fasting beta-cell function did not differ between the groups (Table 1). From OGTT estimates of beta cell function, IGT and T2D had lower IGI_cp, IGI_ins and disposition index than NGT, whereas the adaptation index was decreased only in T2D (Table 1).

### Serum chemerin concentrations

**Fig 1:**
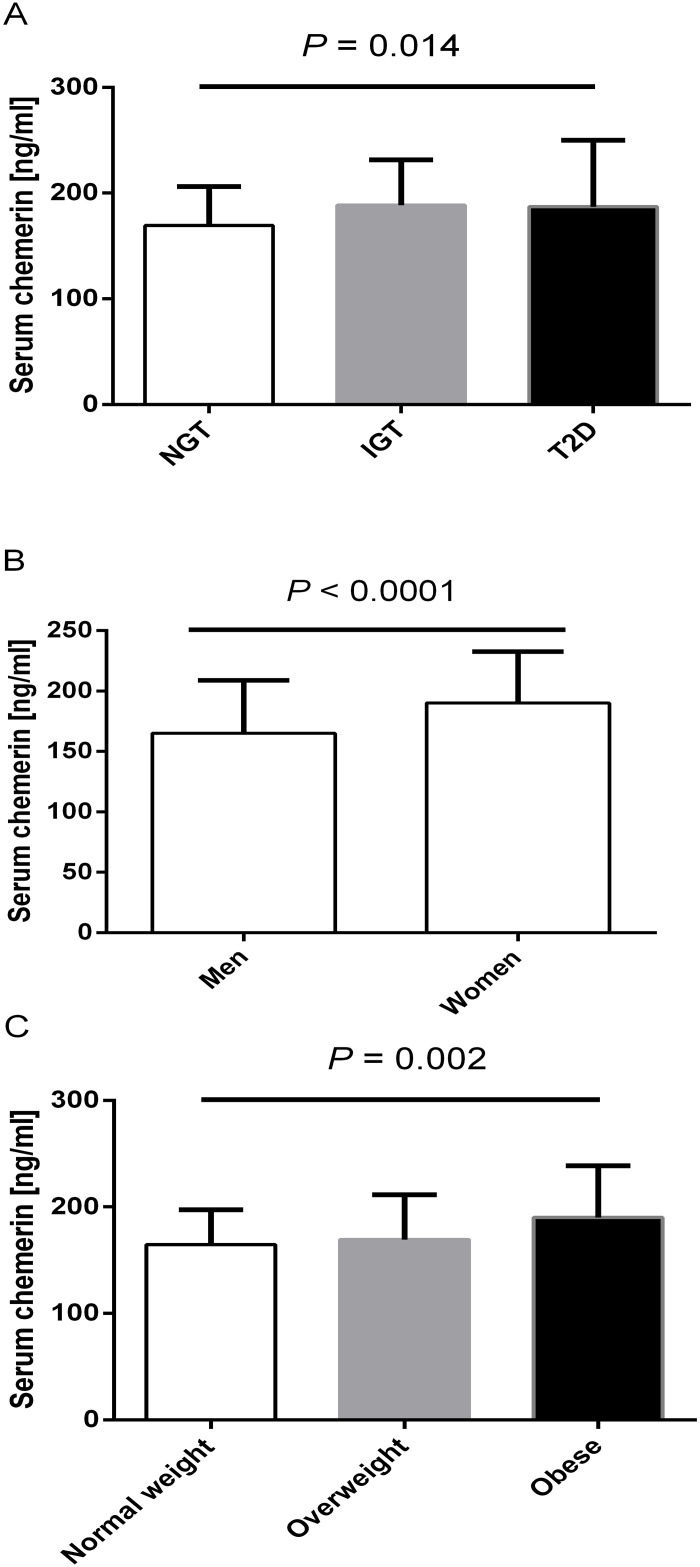
Chemerin serum levels in NAFLD patients (means±SD) stratified by (A) glucose tolerance status (normal (NGT; n = 110) or impaired glucose tolerance (IGT; n = 51) or type 2 diabetes; no sex differences between the 3 groups, P = 0.58), (B) sex and (C) body mass index (normal weight, n = 34; overweight n = 77; obese n = 85).

The three groups differed with regard to serum chemerin levels (*P*_ANOVA_ = 0.014; Fig 1A). Serum chemerin was 169±37 ng/ml in NGT, 189±43 ng/ml in IGT (*P*<0.05 vs. NGT) and 187±63 ng/ml in T2D (*P*>0.05 vs. NGT). Chemerin were 11% higher in the combined IGT+T2D group than in NGT (188±52 ng/ml, *P* = 0.004). Of note, serum chemerin was higher in women than in men (190±42 ng/ml and 165±44 ng/ml, *P*<0.001) (Fig 1B). In addition, serum chemerin levels differed with respect to BMI (*P*_ANOVA_ = 0.002; Fig 1C). Individuals with normal weight, overweight or obesity had serum chemerin levels of 165±33 ng/ml, 169±42 ng/ml (P>0.05 vs. normal weight) and 190±48 ng/ml (P<0.01 vs. normal weight, respectively).

### Associations of chemerin with age, sex and metabolic characteristics

In a joint model containing chemerin, age, sex and BMI, both age and BMI positively associated with serum levels of chemerin, and the sex difference remained significant for chemerin (Table 2). In contrast, no significant associations were found in age-, sex- and BMI-adjusted analyses between chemerin levels and lipids (triglycerides, total, LDL, HDL cholesterol) or transaminases (ALT, AST, γ-GT) (Table 2).

**Table 2:** Associations between Serum Chemerin Levels and Anthropometric and Metabolic Variables.

Variable	Adjusted for age and sex		Adjusted for age, sex and BMI	
	r_s_	*P*	r_s_	*P*
Age*	0.13	0.08	**0.14**	**0.048**
Sex**	**-0.28**	**<0.001**	**-0.26**	**<0.001**
BMI	**0.24**	**<0.001**	**N/A**	**N/A**
**Serum lipids**				
Triglycerides	0.11	0.18	0.06	0.45
Total cholesterol	0.01	0.88	0.00	0.96
HDL cholesterol	-0.15	0.07	-0.10	0.23
LDL cholesterol	0.02	0.81	-0.01	0.93
**Liver enzymes**				
AST	0.04	0.56	-0.01	0.95
ALT	0.10	0.20	0.04	0.60
γ-GT	0.00	0.99	-0.02	0.82
**Measures of glycaemia**				
Fasting glucose	0.04	0.63	-0.02	0.74
2-hr glucose	**0.15**	**0.03**	0.10	0.18
AUC glucose	0.12	0.09	0.06	0.38
Incremental AUC glucose	0.11	0.13	0.06	0.41
**Insulin and C-peptide levels**				
Fasting insulin	**0.17**	**0.02**	0.06	0.39
AUC insulin	0.04	0.58	-0.04	0.60
Incremental AUC insulin	0.01	0.88	-0.06	0.43
Fasting C-peptide	**0.24**	**0.001**	**0.22**	**0.003**
AUC C-peptide	**0.23**	**0.002**	**0.24**	**0.001**
Incremental AUC C-peptide	**0.21**	**0.005**	**0.22**	**0.004**
**Measures of insulin sensitivity**				
QUICKI	**-0.16**	**0.03**	-0.04	0.54
OGIS	-0.06	0.37	0.04	0.58
**Measures of beta-cell function**				
Fasting beta-cell function	**0.24**	**0.001**	**0.23**	**0.002**
Insulinogenic index IGI_ins	-0.08	0.30	-0.10	0.16
Insulinogenic index IGI_cp	**0.18**	**0.02**	**0.20**	**0.007**
Disposition index	-0.04	0.62	-0.06	0.44
Adaptation index	**0.20**	**0.008**	**0.22**	**0.003**
Insulin/C-peptide, molar ratio	**-0.18**	**0.02**	**-0.20**	**0.007**

Data are given as partial Spearman correlation coefficients r_s_ and respective *P* values.

*Adjusted for sex or sex and BMI only.

**Adjusted for age or age and BMI only.

Significant correlations (*P*<0.05) are highlighted using bold print.

In age- and sex-adjusted analyses, serum chemerin levels positively associated with 2-h glucose and fasting insulin, but not with other glucose- and insulin-related variables. After further adjustment for BMI, the significant associations disappeared (Table 2), while serum chemerin associated with C-peptide levels independently of BMI.

Serum chemerin levels neither related to QUICKI nor to OGIS after adjustment for age, sex and BMI. In contrast, serum chemerin associated positively with fasting beta-cell function, IGI_cp as well as adaptation index based on C-peptide, independent of BMI, but not with IGI_ins and disposition index. Serum chemerin also inversely associated with the ratio of insulin to C-peptide (Table 2).

Stratifying the study population into the subgroups with NGT and IGT/T2D did not affect the associations between serum chemerin and metabolic parameters in both subgroups (S1 Table).

## Discussion

This study shows that circulating chemerin concentrations not only positively associate with BMI in patients with NAFLD, but are also higher in individuals with IGT or type 2 diabetes compared to NGT in this study population. Serum chemerin further exhibited BMI-independent associations with beta-cell function, but not with insulin sensitivity.

Chemerin has been proposed as a novel biomarker of insulin resistance related to obesity and T2D, but it was unknown whether the observed associations between chemerin levels and metabolic features are mainly mediated by obesity or occur independently of obesity. In particular, putative associations with T2D are poorly understood because it is not clear whether chemerin primarily relates to insulin resistance or beta-cell dysfunction.

Our study extends previous studies by assessing insulin resistance based on dynamic measurements from an OGTT rather than relying on surrogate markers of insulin resistance derived from fasting parameters only. Although we found that chemerin and QUICKI were correlated, there was no association when OGIS was used as more precise measure of insulin resistance. Moreover, the association with QUICKI was mainly explained by BMI. These data are in line with several studies that also failed to observe a BMI-independent association between chemerin and insulin resistance assessed by HOMA-IR [6,11,14,19,26]. Two previous studies analyzed the association between chemerin and the OGTT-based Matsuda insulin sensitivity index (ISI) [11,26] with contradictory results: the first study in Japanese patients with type 2 diabetes found a significant inverse association after adjustment for age, sex and BMI [11], whereas the inverse association between chemerin and Matsuda ISI in children from Germany was mainly explained by age and BMI [26]. Evidence for a direct BMI-independent association between chemerin and insulin resistance comes from two studies using hyperinsulinemic-normoglycemic clamps in non-obese normoglycemic men [17] and in a very heterogeneous cohort with respect to age, BMI and insulin resistance [27].

The reason for the discrepancy between the aforementioned findings is currently unclear. It is important to note that also mechanistic studies revealed opposing effects of chemerin on insulin action. Kralich et al. showed an increase in insulin—stimulated glucose uptake and insulin receptor substrate-1 tyrosine phosphorylation after chemerin treatment [40], whereas Takahashi et al. reported a decrease in insulin-stimulated glucose uptake [41]. While exogenous administration of chemerin exacerbates glucose intolerance and decreases tissue glucose uptake in obese diabetic mice [42], chemerin-deficient mice are also glucose intolerant [28].

The absence of any correlation between serum chemerin and insulin resistance in the present study could be due to our study population consisting of patients with NAFLD. Several studies indicated that liver disease may affect circulating chemerin levels [42]. Previous studies reported higher chemerin levels in patients with clinical or biopsy-proven NAFLD [13,29,30]. In the present study, patients with IGT and T2D had higher chemerin levels than glucose tolerant patients with NAFLD suggesting a specific role for insulin action or secretion. Nevertheless, a recent study found elevated mRNA levels of both chemerin and its receptor, CMKLR1, in the human liver, with greater expression in patients with NASH [43]. The increase in chemerin in overweight patients with NAFLD may be more pronounced than effects of insulin resistance per se and thereby obscure or blunt any association with insulin resistance. On basis of the previous data and our study, a future study comprising persons without and with NAFLD and careful matching for glucose tolerance status will be necessary to address the question whether the presence of NAFLD modifies the association between chemerin and insulin resistance.

In contrast to insulin resistance, our study clearly showed that chemerin levels associate positively with beta-cell function during fasting and under dynamic conditions as assessed with the insulinogenic index IGI_cp and the adaptation index. These data are novel as two previous studies used only HOMA-B as surrogate marker of fasting beta-cell function. In these studies, chemerin did not relate to HOMA-B in cohorts from Japan [20] and Mauritius [3] after adjustment for age, sex and BMI. Data from studies using dynamic measurements of beta-cell functions have not been published so far. In addition to the fact that dynamic measures are more sensitive to detect changes in insulin secretion, the difference between the present and the previous studies could result form the study population, i.e. Caucasian patients with NAFLD. The generally higher degree of insulin resistance in NAFLD may explain a compensatory excessive increase in insulin secretion during OGTT, which is typical for IGT and early or newly diagnosed patients with T2D [44]. On the other hand, it is conceivable that chemerin per se supports insulin secretion under these conditions. Recent studies on chemerin in mouse models serve to support our human data [28]. Islets from chemerin-deficient mice exhibit impaired glucose-dependent insulin secretion (GSIS), whereas transgenic mice overexpressing chemerin have increased GSIS. Although these data support a beneficial effect of chemerin on beta-cell function, additional preclinical and clinical studies are required to elucidate the interplay between chemerin and insulin secretion in more detail.

The use of dynamic measurements from an OGTT to simultaneously assess insulin sensitivity and beta-cell function represents the main strength of our study. Nevertheless, some limitations should be taken into account. We included patients diagnosed with NAFLD using transaminases and liver ultrasound rather than employing liver biopsy. Although the liver biopsy is the gold standard procedure to diagnose NAFLD [45,46], it is not generally used in mild forms as present in our cohort. Furthermore, we recruited patients form a specialized clinic and did not include persons without NAFLD. Thus, we cannot generalize our findings to the general population or other ethnic groups, but we were able to detect alterations within a well-defined closely matched groups with different degrees of glucose tolerance.

In conclusion, this study did not observe a BMI-independent correlation between increased circulating chemerin concentrations and insulin resistance in patients with NAFLD. On the other hand, we found a robust association between elevated chemerin and increased beta-cell function which points towards a novel beneficial role of chemerin for dynamic insulin secretion in the context of NAFLD and type 2 diabetes.

## Supporting Information

S1 Table Associations between Serum Chemerin Levels and Anthropometric and Metabolic Variables In the Subgroups with NGT and IGT/T2D. (DOCX)
