# Supplementary material for: Serum Chemerin Concentrations Associate with Beta-Cell Function, but Not with Insulin Resistance in Individuals with Non-Alcoholic Fatty Liver Disease (NAFLD)
Source: PLoS One. 2015 May 1;10(5):e0124935. doi: 10.1371/journal.pone.0124935 (PMC4416815; doi:10.1371/journal.pone.0124935)
Supplement: S1 Table — (DOCX) [file pone.0124935.s001.docx]

**Table S1.** Associations between Serum Chemerin Levels and Anthropometric and Metabolic Variables In the Subgroups with NGT and IGT/T2D

| **Variable** | **Adjusted for age and sex** |  |  |  | **Adjusted for age, sex and BMI** |  |  |  |
| --- | --- | --- | --- | --- | --- | --- | --- | --- |
|  | NGT |  | IGT/T2D |  | NGT |  | IGT/T2D |  |
|  | r_s_ | *P* | r_s_ | *P* | r_s_ | *P* | r_s_ | *P* |
| Age* | ‑0.03 | 0.77 | 0.20 | 0.06 | ‑0.01 | 0.94 | **0.22** | **0.04** |
| Sex** | **‑0.24** | **0.01** | **‑0.33** | **0.002** | **‑0.24** | **0.01** | **‑0.29** | **0.007** |
| BMI | **0.28** | **<0.001** | 0.17 | 0.11 | N/A | N/A | N/A | N/A |
| **Serum lipids** |  |  |  |  |  |  |  |  |
| Triglycerides | 0.20 | 0.06 | ‑0.04 | 0.74 | 0.13 | 0.23 | ‑0.05 | 0.69 |
| Total cholesterol | 0.09 | 0.40 | ‑0.01 | 0.92 | 0.10 | 0.35 | ‑0.03 | 0.78 |
| HDL cholesterol | ‑0.14 | 0.20 | ‑0.05 | 0.70 | ‑0.09 | 0.39 | ‑0.02 | 0.87 |
| LDL cholesterol | 0.11 | 0.32 | 0.03 | 0.75 | 0.09 | 0.40 | 0.00 | 1.00 |
| **Liver enzymes** |  |  |  |  |  |  |  |  |
| AST | 0.15 | 0.13 | ‑0.10 | 0.42 | 0.08 | 0.41 | ‑0.12 | 0.32 |
| ALT | 0.10 | 0.32 | 0.07 | 0.55 | 0.02 | 0.84 | 0.04 | 0.70 |
| γ-GT | 0.02 | 0.81 | ‑0.08 | 0.50 | ‑0.03 | 0.80 | ‑0.08 | 0.52 |
| **Measures of glycaemia** |  |  |  |  |  |  |  |  |
| Fasting glucose | ‑0.13 | 0.20 | 0.13 | 0.23 | ‑0.16 | 0.09 | 0.08 | 0.46 |
| 2‑hr glucose | 0.14 | 0.15 | ‑0.06 | 0.61 | 0.09 | 0.35 | ‑0.10 | 0.36 |
| AUC glucose | 0.03 | 0.75 | 0.04 | 0.70 | ‑0.03 | 0.80 | 0.00 | 1.00 |
| Incremental AUC glucose | 0.07 | 0.50 | ‑0.06 | 0.57 | 0.02 | 0.83 | ‑0.09 | 0.43 |
| **Insulin and C-peptide levels** |  |  |  |  |  |  |  |  |
| Fasting insulin | 0.15 | 0.12 | 0.15 | 0.16 | 0.03 | 0.79 | 0.09 | 0.43 |
| AUC insulin | 0.04 | 0.67 | 0.00 | 0.97 | ‑0.04 | 0.66 | ‑0.05 | 0.65 |
| Incremental AUC insulin | 0.03 | 0.77 | ‑0.03 | 0.79 | ‑0.04 | 0.66 | ‑0.08 | 0.49 |
| Fasting C‑peptide | 0.19 | 0.07 | **0.27** | **0.02** | 0.18 | 0.07 | **0.24** | **0.03** |
| AUC C‑peptide | 0.19 | 0.06 | **0.28** | **0.01** | 0.20 | 0.052 | **0.28** | **0.01** |
| Incremental AUC C‑peptide | 0.18 | 0.08 | **0.26** | **0.02** | 0.20 | 0.051 | **0.25** | **0.02** |
| **Measures of insulin sensitivity** |  |  |  |  |  |  |  |  |
| QUICKI | ‑0.10 | 0.28 | ‑0.18 | 0.11 | 0.02 | 0.82 | ‑0.11 | 0.33 |
| OGIS | 0.05 | 0.64 | ‑0.06 | 0.61 | 0.16 | 0.10 | 0.01 | 0.89 |
| **Measures of beta-cell function** |  |  |  |  |  |  |  |  |
| Fasting beta‑cell function | **0.21** | **0.04** | **0.25** | **0.02** | **0.21** | **0.04** | **0.24** | **0.03** |
| Insulinogenic index IGI_ins | ‑0.06 | 0.51 | ‑0.01 | 0.91 | ‑0.10 | 0.28 | ‑0.05 | 0.65 |
| Insulinogenic index IGI_cp | 0.16 | 0.11 | **0.26** | **0.02** | 0.20 | 0.055 | **0.25** | **0.02** |
| Disposition index | 0.06 | 0.54 | ‑0.03 | 0.78 | 0.02 | 0.84 | ‑0.05 | 0.64 |
| Adaptation index | 0.19 | 0.07 | **0.25** | **0.02** | **0.21** | **0.04** | **0.26** | **0.02** |
| Insulin/C‑peptide, molar ratio | ‑0.15 | 0.14 | ‑0.19 | 0.09 | -0.18 | 0.07 | ‑0.20 | 0.07 |

Data are given as partial Spearman correlation coefficients r_s_ and respective *P* values.

*Adjusted for sex or sex and BMI only.

**Adjusted for age or age and BMI only.

Significant correlations (*P*<0.05) are highlighted using bold print.
